# 2-(3-Oxo-1,3-dihydro­isobenzofuran-1-ylamino)benzoic acid^1^

**DOI:** 10.1107/S160053680800754X

**Published:** 2008-03-29

**Authors:** Mustafa Odabaşoğlu, Orhan Büyükgüngör

**Affiliations:** a Department of Chemistry, Faculty of Arts & Science, Ondokuz Mayıs University, TR-55139 Kurupelit Samsun, Turkey; b Department of Physics, Faculty of Arts & Science, Ondokuz Mayıs University, TR-55139 Kurupelit Samsun, Turkey

## Abstract

In the mol­ecule of the title compound, C_15_H_11_NO_4_, the essentially planar phthalide group is oriented at a dihedral angle of 56.78 (5)° with respect to the substituted aromatic ring. An intra­molecular N—H⋯O hydrogen bond results in the formation of a non-planar six-membered ring, which adopts a nearly flattened-boat conformation. In the crystal structure, inter­molecular C—H⋯O, O—H⋯O and N—H⋯O hydrogen bonds link the mol­ecules, generating centrosymmetric *R*_2_^2^(8) and *R*_2_^2^(11) ring motifs and forming a three-dimensional network.

## Related literature

For general background, see: Aoki *et al.* (1973 ▶, 1974 ▶); Lacova (1973 ▶, 1974 ▶); Elderfield (1951 ▶); Bellasio (1974 ▶, 1975 ▶); Roy & Sarkar (2005 ▶); Kubota & Tatsuno (1971 ▶); Tsi & Tan (1997 ▶). For related structures, see: Büyükgüngör & Odabaşoğlu (2006 ▶); Odabaşoğlu & Büyükgüngör (2006 ▶; 2007 ▶). For ring puckering parameters, see: Cremer & Pople (1975 ▶). For ring motif details, see: Bernstein *et al.* (1995 ▶); Etter (1990 ▶).
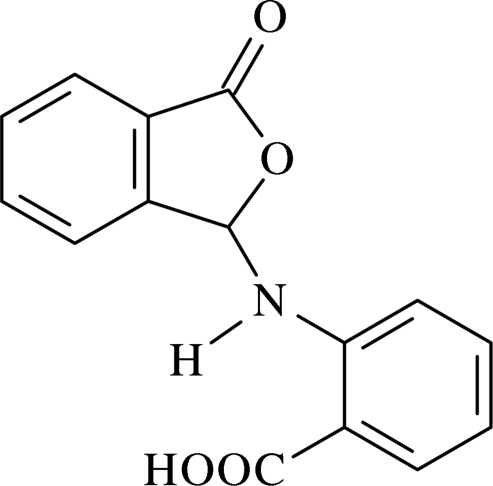


## Experimental

### 

#### Crystal data

C_15_H_11_NO_4_
*M* *_r_* = 269.25
Monoclinic, 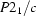

*a* = 7.8135 (6) Å
*b* = 22.6205 (10) Å
*c* = 7.0902 (5) Å
β = 101.061 (5)°
*V* = 1229.88 (14) Å^3^
*Z* = 4
Mo *K*α radiation
μ = 0.11 mm^−1^
*T* = 296 K
0.55 × 0.36 × 0.18 mm

#### Data collection

Stoe IPDS II diffractometer
Absorption correction: integration (*X-RED32*; Stoe & Cie, 2002 ▶) *T*_min_ = 0.958, *T*_max_ = 0.982
12715 measured reflections
2536 independent reflections
1958 reflections with *I* > 2σ(*I*)
*R*_int_ = 0.034

#### Refinement

*R*[*F* ^2^ > 2σ(*F* ^2^)] = 0.043
*wR*(*F* ^2^) = 0.093
*S* = 1.07
2536 reflections
225 parameters
All H-atom parameters refined
Δρ_max_ = 0.16 e Å^−3^
Δρ_min_ = −0.17 e Å^−3^

### 

Data collection: *X-AREA* (Stoe & Cie, 2002 ▶); cell refinement: *X-AREA*; data reduction: *X-RED32* (Stoe & Cie, 2002 ▶); program(s) used to solve structure: *SHELXS97* (Sheldrick, 2008 ▶); program(s) used to refine structure: *SHELXL97* (Sheldrick, 2008 ▶); molecular graphics: *ORTEP-3 for Windows* (Farrugia, 1997 ▶); software used to prepare material for publication: *WinGX* (Farrugia, 1999 ▶).

## Supplementary Material

Crystal structure: contains datablocks I, global. DOI: 10.1107/S160053680800754X/hk2434sup1.cif

Structure factors: contains datablocks I. DOI: 10.1107/S160053680800754X/hk2434Isup2.hkl

Additional supplementary materials:  crystallographic information; 3D view; checkCIF report

## Figures and Tables

**Table 1:** Hydrogen-bond geometry (Å, °)

*D*—H⋯*A*	*D*—H	H⋯*A*	*D*⋯*A*	*D*—H⋯*A*
N1—H1⋯O3	0.86 (2)	2.074 (19)	2.7004 (18)	129.3 (16)
N1—H1⋯O1^i^	0.86 (2)	2.58 (2)	3.281 (2)	138.9 (15)
O4—H4*A*⋯O3^ii^	0.97 (3)	1.67 (3)	2.6329 (17)	174 (2)
C4—H4⋯O2^iii^	0.93 (2)	2.58 (2)	3.464 (2)	158.9 (17)
C8—H8⋯O1^iv^	0.983 (18)	2.580 (17)	3.403 (2)	141.4 (12)

Symmetry codes: (i) 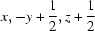; (ii) 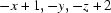; (iii) 
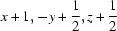
; (iv) 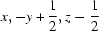.

**Fig. 1.**
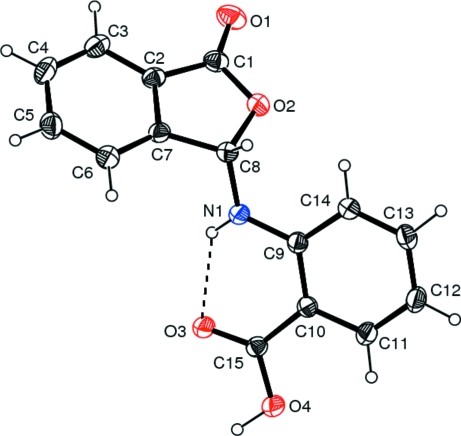
The molecular structure of the title molecule, with the atom-numbering scheme. Displacement ellipsoids are drawn at the 30% probability level. Hydrogen bond is shown as dashed line.

**Fig. 2.**
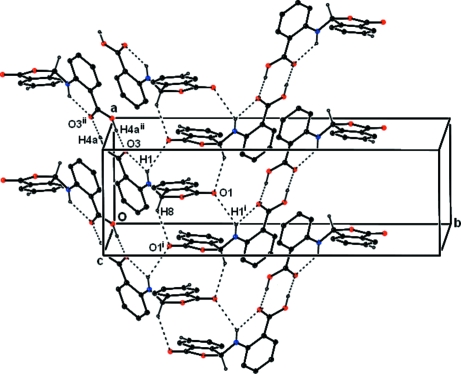
A partial packing diagram of (I), showing the formation of R22(8) and R22(11) ring motifs. Hydrogen bonds are shown as dashed lines [symmetry codes: (i) x, 1/2 - y, z - 1/2; (ii) 1 - x, -y, 2 - z]. H atoms not involved in hydrogen bondings have been omitted for clarity.

## supplementary crystallographic information

### Comment

Phthalides are known to show diverse biological activities as hormones, pheromones and antibiotics (Aoki *et al.*,  1973, 1974; Lacova, 1973, 1974; Elderfield, 1951; Bellasio, 1974, 1975; Roy & Sarkar, 2005; Kubota & Tatsuno, 1971; Tsi & Tan, 1997). As part of our ongoing research on 3-substituted phthalides (Büyükgüngör & Odabaşoğlu, 2006; Odabaşoğlu & Büyükgüngör, 2006; 2007), the title compound, (I), has been synthesized and its crystal structure is reported here.

In the molecule of (I), (Fig. 1), rings A (C2-C7), B (C1/C2/C7/C8/O2) and C (C9-C14) are, of course, planar. The dihedral angles between them are A/B = 3.08 (3)°, A/C = 57.11 (4)° and B/C = 56.56 (5)°. So, rings A and B are also nearly coplanar. Ring C is oriented with respect to the coplanar ring system at a dihedral angle of 56.78 (5)°. The intramolecular N-H···O hydrogen bond (Table 1) results in the formation of a non-planar six-membered ring D (N1/H1/O3/C9/C10/C15), having total puckering amplitude, Q_T_, of 1.408 (3) Å, in which it adopts a nearly flattened-boat [φ = -42.43 (2)° and θ = 97.94 (3)°] conformation (Cremer & Pople, 1975).

In the crystal structure, intermolecular C-H···O, O-H···O and N-H···O hydrogen bonds (Table 1) link the molecules, generating centrosymmetric R_2_^2^(8) and R_2_^2^(11) (Fig. 2) ring motifs (Bernstein *et al.*,  1995; Etter, 1990), to form a three-dimensional network, in which they may be effective in the stabilization of the structure.

### Experimental

The title compound was prepared according to the method described by Odabaşoğlu & Büyükgüngör (2006), using phthalaldehydic acid and antranilic acid as starting materials (yield; 70%). Crystals of (I) suitable for X-ray analysis were obtained by slow evaporation of an ethanol-DMF (1:1) solution at room temperature.

### Refinement

H atoms were located in difference synthesis and refined freely [C-H = 0.93 (2)-0.983 (18) Å and U_iso_(H) = 0.036 (4)-0.060 (6) Å^2^; N-H = 0.86 (2) Å and U_iso_(H) = 0.046 (5) Å^2^; O-H = 0.97 (3) Å and U_iso_(H) = 0.094 (9) Å^2^].

### Crystal data

**Table:**

C_15_H_11_NO_4_	*F*_000_ = 560
*M**_r_* = 269.25	*D*_x_ = 1.454 Mg m^−^^3^
Monoclinic, *P*2_1_/*c*	Mo *K*α radiation λ = 0.71073 Å
Hall symbol: -P 2ybc	Cell parameters from 12715 reflections
*a* = 7.8135 (6) Å	θ = 1.8–27.3º
*b* = 22.6205 (10) Å	µ = 0.11 mm^−^^1^
*c* = 7.0902 (5) Å	*T* = 296 K
β = 101.061 (5)º	Prism, colorless
*V* = 1229.88 (14) Å^3^	0.55 × 0.36 × 0.18 mm
*Z* = 4	

### Data collection

**Table:**

Stoe IPDSII diffractometer	2536 independent reflections
Monochromator: plane graphite	1958 reflections with *I* > 2σ(*I*)
Detector resolution: 6.67 pixels mm^-1^	*R*_int_ = 0.034
*T* = 296 K	θ_max_ = 26.5º
ω scan rotation method	θ_min_ = 1.8º
Absorption correction: integration(X-RED32; Stoe & Cie, 2002)	*h* = −9→9
*T*_min_ = 0.958, *T*_max_ = 0.982	*k* = −28→28
12715 measured reflections	*l* = −8→8

### Refinement

**Table:**

Refinement on *F*^2^	Secondary atom site location: difference Fourier map
Least-squares matrix: full	Hydrogen site location: inferred from neighbouring sites
*R*[*F*^2^ > 2σ(*F*^2^)] = 0.043	All H-atom parameters refined
*wR*(*F*^2^) = 0.093	*w* = 1/[σ^2^(*F*_o_^2^) + (0.0403*P*)^2^ + 0.2251*P*] where *P* = (*F*_o_^2^ + 2*F*_c_^2^)/3
*S* = 1.07	(Δ/σ)_max_ < 0.001
2536 reflections	Δρ_max_ = 0.16 e Å^−^^3^
225 parameters	Δρ_min_ = −0.17 e Å^−^^3^
Primary atom site location: structure-invariant direct methods	Extinction correction: none

### Special details

**Table:**

Geometry. All e.s.d.'s (except the e.s.d. in the dihedral angle between two l.s. planes) are estimated using the full covariance matrix. The cell e.s.d.'s are taken into account individually in the estimation of e.s.d.'s in distances, angles and torsion angles; correlations between e.s.d.'s in cell parameters are only used when they are defined by crystal symmetry. An approximate (isotropic) treatment of cell e.s.d.'s is used for estimating e.s.d.'s involving l.s. planes.
Refinement. Refinement of F^2^ against ALL reflections. The weighted R-factor wR and goodness of fit S are based on F^2^, conventional R-factors R are based on F, with F set to zero for negative F^2^. The threshold expression of F^2^ > 2sigma(F^2^) is used only for calculating R-factors(gt) etc. and is not relevant to the choice of reflections for refinement. R-factors based on F^2^ are statistically about twice as large as those based on F, and R- factors based on ALL data will be even larger.

### Fractional atomic coordinates and isotropic or equivalent isotropic displacement parameters (Å^2^)

**Table:**

	*x*	*y*	*z*	*U*_iso_*/*U*_eq_	
C1	0.6576 (2)	0.26649 (7)	0.4899 (2)	0.0402 (4)	
C2	0.8303 (2)	0.24430 (7)	0.5812 (2)	0.0367 (4)	
C3	0.9781 (3)	0.27532 (8)	0.6650 (3)	0.0457 (4)	
C4	1.1272 (3)	0.24364 (9)	0.7337 (3)	0.0518 (5)	
C5	1.1279 (3)	0.18248 (9)	0.7191 (3)	0.0511 (5)	
C6	0.9805 (2)	0.15141 (8)	0.6356 (3)	0.0460 (4)	
C7	0.8308 (2)	0.18333 (7)	0.5662 (2)	0.0354 (4)	
C8	0.6571 (2)	0.16362 (7)	0.4548 (3)	0.0364 (4)	
C9	0.4218 (2)	0.09230 (6)	0.4534 (2)	0.0332 (4)	
C10	0.3436 (2)	0.04653 (6)	0.5436 (2)	0.0321 (3)	
C11	0.1982 (2)	0.01723 (7)	0.4399 (3)	0.0396 (4)	
C12	0.1264 (2)	0.03208 (8)	0.2541 (3)	0.0458 (4)	
C13	0.2021 (2)	0.07689 (8)	0.1666 (3)	0.0435 (4)	
C14	0.3465 (2)	0.10673 (7)	0.2628 (2)	0.0389 (4)	
C15	0.4117 (2)	0.02788 (6)	0.7427 (2)	0.0334 (3)	
N1	0.56924 (19)	0.12145 (6)	0.5475 (2)	0.0387 (3)	
O1	0.60404 (19)	0.31643 (5)	0.4713 (2)	0.0563 (4)	
O2	0.55684 (15)	0.21973 (5)	0.41990 (18)	0.0437 (3)	
O3	0.53512 (17)	0.05196 (5)	0.84778 (17)	0.0457 (3)	
O4	0.32808 (17)	−0.01696 (5)	0.80153 (19)	0.0481 (3)	
H1	0.596 (2)	0.1184 (8)	0.670 (3)	0.046 (5)*	
H3	0.975 (3)	0.3188 (9)	0.671 (3)	0.058 (6)*	
H4	1.229 (3)	0.2630 (9)	0.792 (3)	0.060 (6)*	
H4A	0.383 (3)	−0.0277 (11)	0.931 (4)	0.094 (9)*	
H5	1.233 (3)	0.1623 (8)	0.764 (3)	0.055 (6)*	
H6	0.980 (3)	0.1090 (9)	0.626 (3)	0.053 (5)*	
H8	0.665 (2)	0.1498 (7)	0.325 (3)	0.036 (4)*	
H11	0.149 (2)	−0.0136 (8)	0.506 (3)	0.045 (5)*	
H12	0.030 (3)	0.0113 (9)	0.191 (3)	0.055 (6)*	
H13	0.154 (2)	0.0893 (8)	0.035 (3)	0.049 (5)*	
H14	0.394 (2)	0.1377 (8)	0.199 (3)	0.047 (5)*	

### Atomic displacement parameters (Å^2^)

**Table:**

	*U*^11^	*U*^22^	*U*^33^	*U*^12^	*U*^13^	*U*^23^
C1	0.0518 (10)	0.0365 (8)	0.0337 (9)	−0.0007 (7)	0.0115 (8)	0.0015 (6)
C2	0.0436 (9)	0.0365 (8)	0.0316 (8)	−0.0055 (7)	0.0113 (7)	−0.0002 (6)
C3	0.0524 (11)	0.0441 (10)	0.0421 (10)	−0.0141 (8)	0.0130 (8)	−0.0057 (8)
C4	0.0421 (11)	0.0669 (12)	0.0456 (11)	−0.0212 (10)	0.0068 (9)	−0.0065 (9)
C5	0.0371 (10)	0.0647 (12)	0.0497 (11)	0.0009 (9)	0.0041 (9)	0.0057 (9)
C6	0.0433 (10)	0.0409 (9)	0.0520 (11)	0.0000 (8)	0.0040 (8)	0.0030 (8)
C7	0.0375 (9)	0.0359 (8)	0.0333 (9)	−0.0051 (7)	0.0080 (7)	0.0024 (6)
C8	0.0389 (9)	0.0318 (8)	0.0373 (9)	−0.0013 (7)	0.0040 (7)	0.0031 (6)
C9	0.0321 (8)	0.0308 (7)	0.0361 (9)	0.0020 (6)	0.0044 (7)	−0.0020 (6)
C10	0.0298 (8)	0.0316 (7)	0.0351 (9)	0.0028 (6)	0.0066 (7)	0.0001 (6)
C11	0.0329 (9)	0.0422 (9)	0.0429 (10)	−0.0047 (7)	0.0054 (7)	−0.0011 (7)
C12	0.0366 (10)	0.0522 (10)	0.0448 (11)	−0.0082 (8)	−0.0017 (8)	−0.0046 (8)
C13	0.0426 (10)	0.0487 (10)	0.0351 (10)	0.0033 (8)	−0.0025 (8)	0.0010 (7)
C14	0.0400 (10)	0.0378 (8)	0.0375 (9)	−0.0014 (7)	0.0041 (7)	0.0041 (7)
C15	0.0316 (8)	0.0309 (7)	0.0385 (9)	−0.0003 (6)	0.0089 (7)	−0.0008 (6)
N1	0.0415 (8)	0.0396 (7)	0.0325 (8)	−0.0098 (6)	0.0010 (6)	0.0048 (6)
O1	0.0735 (10)	0.0368 (6)	0.0577 (9)	0.0114 (6)	0.0107 (7)	0.0040 (6)
O2	0.0425 (7)	0.0373 (6)	0.0479 (7)	0.0009 (5)	−0.0001 (6)	0.0049 (5)
O3	0.0525 (8)	0.0435 (6)	0.0369 (7)	−0.0141 (6)	−0.0020 (6)	0.0051 (5)
O4	0.0461 (7)	0.0540 (7)	0.0419 (7)	−0.0159 (6)	0.0028 (6)	0.0113 (6)

### Geometric parameters (Å, °)

**Table:**

O4—H4A	0.97 (3)	C8—O2	1.4872 (18)
N1—H1	0.86 (2)	C8—H8	0.983 (18)
C1—O1	1.203 (2)	C9—N1	1.383 (2)
C1—O2	1.355 (2)	C9—C14	1.405 (2)
C1—C2	1.469 (2)	C9—C10	1.415 (2)
C2—C7	1.383 (2)	C10—C11	1.397 (2)
C2—C3	1.384 (2)	C10—C15	1.472 (2)
C3—C4	1.375 (3)	C11—C12	1.371 (3)
C3—H3	0.98 (2)	C11—H11	0.96 (2)
C4—C5	1.387 (3)	C12—C13	1.380 (3)
C4—H4	0.93 (2)	C12—H12	0.93 (2)
C5—C6	1.382 (3)	C13—C14	1.378 (2)
C5—H5	0.94 (2)	C13—H13	0.98 (2)
C6—C7	1.382 (2)	C14—H14	0.948 (19)
C6—H6	0.963 (19)	C15—O3	1.2271 (19)
C7—C8	1.501 (2)	C15—O4	1.3168 (19)
C8—N1	1.409 (2)		
			
C1—O2—C8	110.76 (12)	N1—C8—C7	115.34 (14)
C15—O4—H4A	109.6 (15)	O2—C8—C7	103.22 (12)
C9—N1—C8	122.22 (14)	N1—C8—H8	110.1 (10)
C9—N1—H1	118.1 (13)	O2—C8—H8	104.0 (10)
C8—N1—H1	118.7 (13)	C7—C8—H8	111.9 (10)
O1—C1—O2	121.82 (16)	N1—C9—C14	120.64 (15)
O1—C1—C2	129.81 (16)	N1—C9—C10	121.49 (14)
O2—C1—C2	108.36 (13)	C14—C9—C10	117.86 (14)
C7—C2—C3	121.61 (16)	C11—C10—C9	119.13 (15)
C7—C2—C1	108.84 (14)	C11—C10—C15	118.49 (14)
C3—C2—C1	129.51 (15)	C9—C10—C15	122.37 (14)
C4—C3—C2	117.92 (17)	C12—C11—C10	122.10 (16)
C4—C3—H3	122.0 (12)	C12—C11—H11	121.2 (11)
C2—C3—H3	120.0 (12)	C10—C11—H11	116.7 (11)
C3—C4—C5	120.56 (18)	C11—C12—C13	118.70 (16)
C3—C4—H4	120.3 (13)	C11—C12—H12	119.0 (12)
C5—C4—H4	119.1 (13)	C13—C12—H12	122.3 (13)
C6—C5—C4	121.62 (19)	C14—C13—C12	121.22 (17)
C6—C5—H5	120.1 (12)	C14—C13—H13	117.3 (11)
C4—C5—H5	118.3 (12)	C12—C13—H13	121.5 (11)
C5—C6—C7	117.75 (17)	C13—C14—C9	120.98 (16)
C5—C6—H6	122.0 (12)	C13—C14—H14	118.8 (11)
C7—C6—H6	120.3 (12)	C9—C14—H14	120.2 (11)
C6—C7—C2	120.54 (16)	O3—C15—O4	121.99 (15)
C6—C7—C8	130.55 (15)	O3—C15—C10	123.52 (14)
C2—C7—C8	108.71 (14)	O4—C15—C10	114.49 (13)
N1—C8—O2	111.53 (14)		
			
O1—C1—O2—C8	177.50 (16)	N1—C8—O2—C1	127.43 (15)
C2—C1—O2—C8	−1.67 (18)	C7—C8—O2—C1	3.01 (18)
O1—C1—C2—C7	−179.60 (18)	O2—C8—N1—C9	72.79 (19)
O2—C1—C2—C7	−0.52 (19)	C7—C8—N1—C9	−169.91 (14)
O1—C1—C2—C3	−2.1 (3)	C14—C9—N1—C8	−4.3 (2)
O2—C1—C2—C3	177.00 (17)	C10—C9—N1—C8	174.57 (15)
C7—C2—C3—C4	0.0 (3)	N1—C9—C10—C11	−177.90 (15)
C1—C2—C3—C4	−177.23 (17)	C14—C9—C10—C11	1.0 (2)
C2—C3—C4—C5	−0.1 (3)	N1—C9—C10—C15	1.1 (2)
C3—C4—C5—C6	0.1 (3)	C14—C9—C10—C15	−179.97 (15)
C4—C5—C6—C7	0.0 (3)	C9—C10—C11—C12	−1.2 (2)
C5—C6—C7—C2	−0.1 (3)	C15—C10—C11—C12	179.71 (16)
C5—C6—C7—C8	174.15 (19)	C10—C11—C12—C13	0.9 (3)
C3—C2—C7—C6	0.1 (3)	C11—C12—C13—C14	−0.4 (3)
C1—C2—C7—C6	177.86 (16)	C12—C13—C14—C9	0.3 (3)
C3—C2—C7—C8	−175.31 (16)	N1—C9—C14—C13	178.36 (16)
C1—C2—C7—C8	2.45 (19)	C10—C9—C14—C13	−0.5 (2)
C6—C7—C8—N1	60.0 (3)	C11—C10—C15—O3	−177.61 (16)
C2—C7—C8—N1	−125.16 (15)	C9—C10—C15—O3	3.4 (2)
C6—C7—C8—O2	−178.07 (18)	C11—C10—C15—O4	1.8 (2)
C2—C7—C8—O2	−3.27 (18)	C9—C10—C15—O4	−177.24 (14)

### Hydrogen-bond geometry (Å, °)

**Table:**

*D*—H···*A*	*D*—H	H···*A*	*D*···*A*	*D*—H···*A*
N1—H1···O3	0.86 (2)	2.074 (19)	2.7004 (18)	129.3 (16)
N1—H1···O1^i^	0.86 (2)	2.58 (2)	3.281 (2)	138.9 (15)
O4—H4A···O3^ii^	0.97 (3)	1.67 (3)	2.6329 (17)	174 (2)
C4—H4···O2^iii^	0.93 (2)	2.58 (2)	3.464 (2)	158.9 (17)
C8—H8···O1^iv^	0.983 (18)	2.580 (17)	3.403 (2)	141.4 (12)

Symmetry codes: (i) *x*, −*y*+1/2, *z*+1/2; (ii) −*x*+1, −*y*, −*z*+2; (iii) *x*+1, −*y*+1/2, *z*+1/2; (iv) *x*, −*y*+1/2, *z*−1/2.
